# AGK enhances angiogenesis and inhibits apoptosis via activation of the NF-κB signaling pathway in hepatocellular carcinoma

**DOI:** 10.18632/oncotarget.2666

**Published:** 2014-11-29

**Authors:** Yanmei Cui, Chuyong Lin, Zhiqiang Wu, Aibin Liu, Xin Zhang, Jinrong Zhu, Geyan Wu, Jueheng Wu, Mengfeng Li, Jun Li, Libing Song

**Affiliations:** ^1^ State Key Laboratory of Oncology in Southern China, Department of Experimental Research, Collaborative Innovation Center for Cancer Medicine, Sun Yat-sen University Cancer Center, Guangzhou 510060, China; ^2^ Department of Biochemistry, Zhongshan School of Medicine, Sun Yat-sen University, Guangzhou, Guangdong 510080, China; ^3^ Department of Microbiology, Zhongshan School of Medicine, Sun Yat-sen University, Guangzhou, Guangdong 510080, China

**Keywords:** AGK, Hepatocellular carcinoma, Angiogenesis, Apoptosis, NF-κB signaling

## Abstract

High levels of angiogenesis and resistance to apoptosis are major clinical features of hepatocellular carcinoma (HCC), a lethal disease with a high incidence worldwide. However, the precise mechanisms underlying these malignant properties remain unclear. Here, we demonstrated that acylglycerol kinase (AGK) is markedly overexpressed in HCC cell lines and clinical tissues. Immunohistochemical analysis of 245 clinical HCC specimens revealed patients with high levels of AGK expression had poorer overall survival compared to patients with low AGK expression. Furthermore, overexpressing AGK significantly enhanced angiogenesis and inhibited apoptosis *in vitro* and promoted the tumorigenicity of HCC cells *in vivo*; silencing endogenous AGK had the opposite effects. Importantly, AGK enhanced angiogenesis and inhibited apoptosis in HCC in part via activation of NF-κB signaling. Our findings provide new evidence that AGK plays an important role in promoting angiogenesis and providing resistance to apoptosis, thus AGK may represent a novel therapeutic target for HCC.

## INTRODUCTION

Hepatocellular carcinoma (HCC) is one of the most common types of cancer and fourth leading cause of cancer-related deaths worldwide [1]. HCC is characterized by a progressive pattern of development and has an extremely poor prognosis; both of these characteristics have been attributed to the highly vascular nature of HCC [2, 3]. Vascular endothelial growth factor (VEGF) has been identified as a major proangiogenic factor and independent prognostic marker in HCC [4–6]. Additionally, HCC cells proliferate rapidly and are highly resistant to apoptosis, resulting in significant insensitivity to chemotherapy [7]. Therefore, investigation of the mechanisms that contribute to angiogenesis and resistance to apoptosis may help to further uncover the biological basis of HCC and improve clinical therapy [8–11].

The nuclear factor-κB (NF-κB) pathway plays important roles in the regulation of angiogenesis and cell survival and is constitutively activated in a variety of human cancers, including HCC [12–16]. Upon stimulation by extracellular signals, the inhibitors of NF-κB (IκBs) could be phosphorylated by IκB kinase (IKK) and subsequently ubiquitinated, leading to proteasomal degradation of IκBs and translocation of cytoplasmic NF-κB p50/p65 into the nucleus, thereby activating the transcription of various NF-κB target genes, including *Bcl-X_L_, XIAP, FLIP* and *VEGF*, which protect against apoptosis and promote angiogenesis [12, 17, 18]. Recently, mutations in components of the NF-κB signaling system have been identified in multiple hematopoietic malignancies and are thought to result in cell-antonomous activation of NF-κB; however, extensive research has failed to identify NF-κB-activating mutations in most solid tumor types including HCC [17, 19–21]. Therefore, identification of the causes that lead to aberrant activation of the NF-κB pathway in HCC is urgently required.

Acylglycerol kinase (AGK) was originally identified as a multisubstrate lipid kinase, and catalyzes the phosphorylation of acylglycerols to generate lysophosphatidic acid (LPA), thus regulating multiple cellular processes related to pathogenesis of cancer [22]. AGK has been reported to be upregulated in various tumor types including prostate cancer, esophageal squamous cell carcinoma, ovarian cancer, gastric cancer and breast cancer [23–26]. Overexpression of AGK transactivates epidermal growth factor receptor (EGFR) and enhances the proliferation and migration of prostate cancer cells *in vitro* [23, 24, 27]. However, the clinical implications and function of AGK in HCC are not well defined.

In the present study, we report that AGK is significantly upregulated in HCC and correlated with poorer overall survival in patients with HCC. Moreover, AGK enhanced angiogenesis and inhibited apoptosis in HCC cells both *in vitro* and *in vivo*, at least in part via activation of the NF-κB signaling pathway. This study reveals a novel mechanism that may contribute to the poor prognosis of HCC and may also provide a novel therapeutic target.

## RESULTS

### AGK is upregulated and is associated with a poor prognosis in HCC

**Figure 1:**
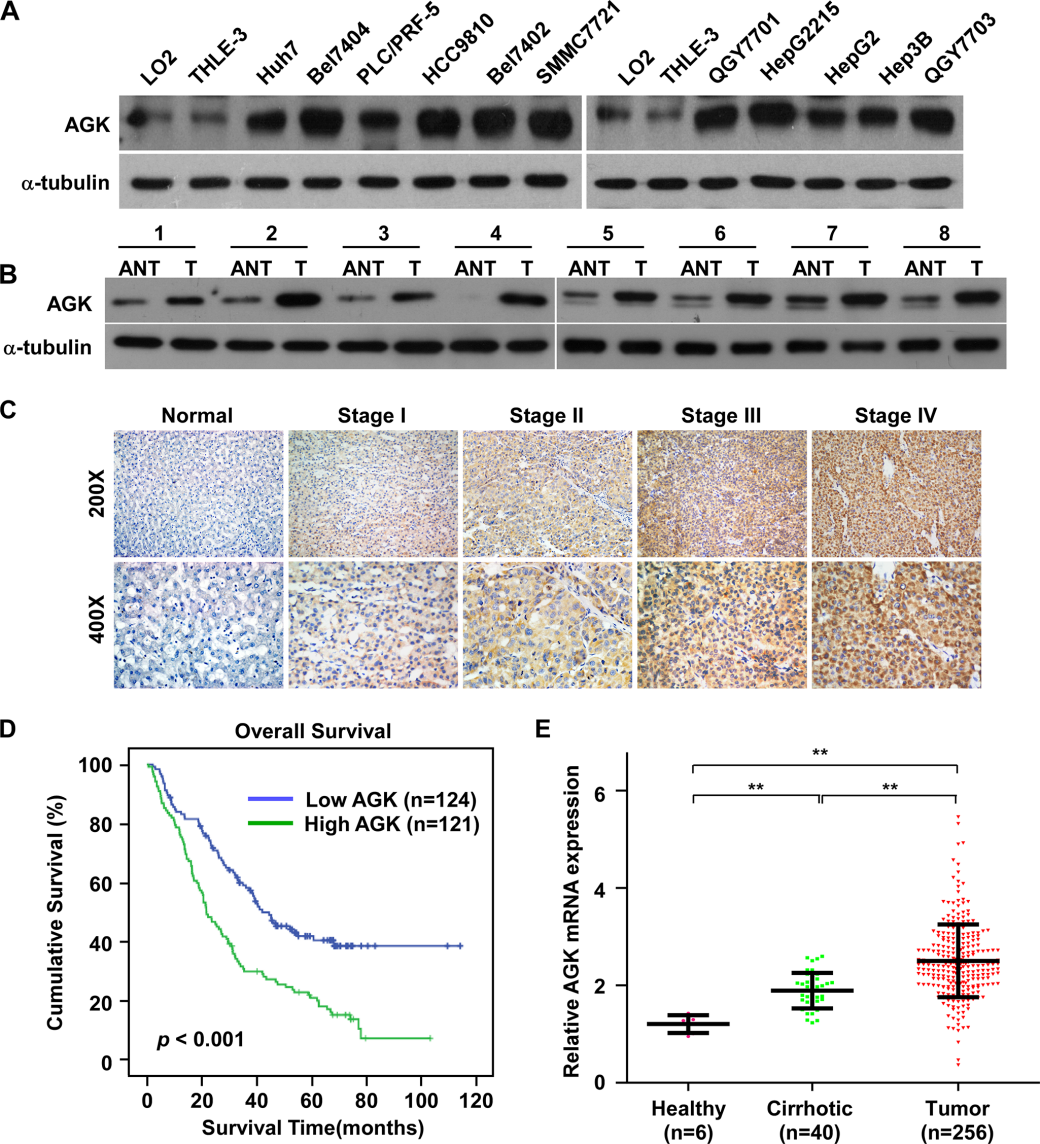
AGK is upregulated in HCC cell lines and primary human HCC tissues **(A and B)** Western blotting analysis of AGK expression in two immortalized normal liver cell lines and 11 HCC cell lines **(A)** and in eight paired primary HCC tissues (*T*) and the adjacent noncancerous tissues (*ANT*) from the same patients **(B)**; α-tubulin was used as a loading control. **(C)** IHC staining of AGK expression in human HCC (clinical stages I–IV) and normal liver tissues. **(D)** Kaplan-Meier overall survival curves for patients with HCC stratified by low (*n* = 124) and high (*n* = 121) expression of AGK (*P* < 0.001). **(E)** GEO data (GSE25097) analysis of *AGK* mRNA expression in normal liver tissues from healthy individuals (*n* = 6), cirrhotic tissues (*n* = 40) and HCC tissues (*n* = 256); **P < 0.001.

To investigate the role of AGK in the progression of HCC, we first examined the expression pattern of AGK in HCC cell lines and human HCC tissues. Real-time PCR and western blotting analyses showed that AGK mRNA and protein expression were significantly upregulated in all eleven HCC cell lines tested compared to two immortalized normal liver cell lines (Figure 1A and Supplementary Figure 1A). Moreover, AGK expression was markedly upregulated in eight HCC tissues compared to the paired adjacent noncancerous tissues (Figure 1B and Supplementary Figure 1B). Collectively, these data demonstrate that AGK is upregulated in HCC.

To investigate the clinical significance of upregulation of AGK in HCC, AGK protein expression was examined in 245 paraffin-embedded, archived HCC tissues using immunohistochemistry (IHC). As shown in Figure 1C and Supplementary Table 1, expression of AGK correlated significantly with clinical stage (*P* < 0.001) and T classification (*P* = 0.001) in HCC. Notably, Kaplan-Meier survival analysis revealed that patients with high AGK expression had poorer overall survival than patients with low AGK expression (Figure 1D, *P* < 0.001), indicating that AGK may have potential as an independent prognostic marker in HCC. To further confirm the clinicopathological relevance of AGK in HCC, we analyzed *AGK* mRNA expression in published expression profiles from GEO dataset (GSE25097). Interestingly, the expression of *AGK* sequentially increased in healthy liver, cirrhosis tissues and HCC (Figure 1E), suggesting that AGK may play a crucial role in the pathogenesis of HCC.

### AGK promotes an aggressive phenotype in HCC cells *in vitro*

**Figure 2:**
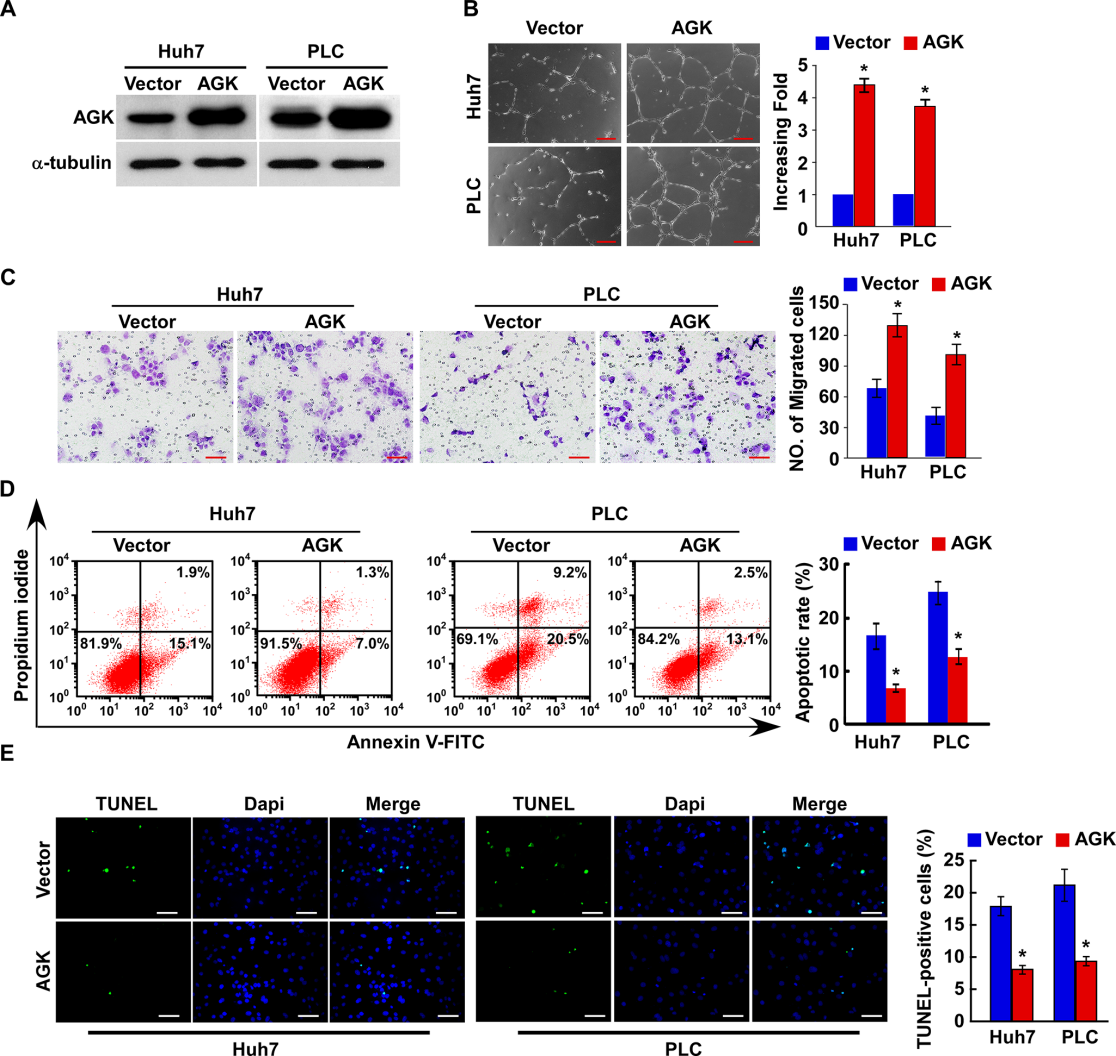
Overexpression of AGK promotes angiogenesis and inhibits apoptosis in HCC cells *in vitro* **(A)** Overexpression of AGK in Huh7 and PLC cell lines was confirmed by western blotting; α-tubulin was used as a loading control. **(B)** Representative images (left) and quantification (right) of tubule formation by HUVECs cultured on Matrigel-coated plates with conditioned medium collected from the indicated HCC cells. Scale bars: 200 μm. Each bar represents the mean ± SD of three independent experiments; *P < 0.05. **(C)** Cell migration was assessed by culturing HUVECs with conditioned media collected from the indicated HCC cells. Scale bars: 50 μm. Each bar represents the mean ± SD of three independent experiments; *P < 0.05. **(D)** Annexin V-FITC/PI staining of the indicated cells after treatment with cisplatin (20 μM) for 24 hours. Each bar represents the mean ± SD of three independent experiments; *P < 0.05. **(E)** Representative images (left) and quantification of TUNEL-positive cells in the indicated cells after treatment with cisplatin (20 μM) for 24 hours. Scale bars: 50 μm. Each bar represents the mean ± SD of three independent experiments; *P < 0.05.

To investigate the biological function of AGK during the pathogenesis of HCC, we established stable AGK-expressing HCC cells using the cell lines Huh-7 and PLC (Figure 2A). Strikingly, overexpression of AGK strongly enhanced the ability of HCC cells to induce tubule formation and migration by human umbilical vein endothelial cells (HUVECs) (Figure 2B and C). Moreover, overexpression of AGK conferred resistance to cisplatin-induced apoptosis in HCC cells (Figure 2D and E).

**Figure 3:**
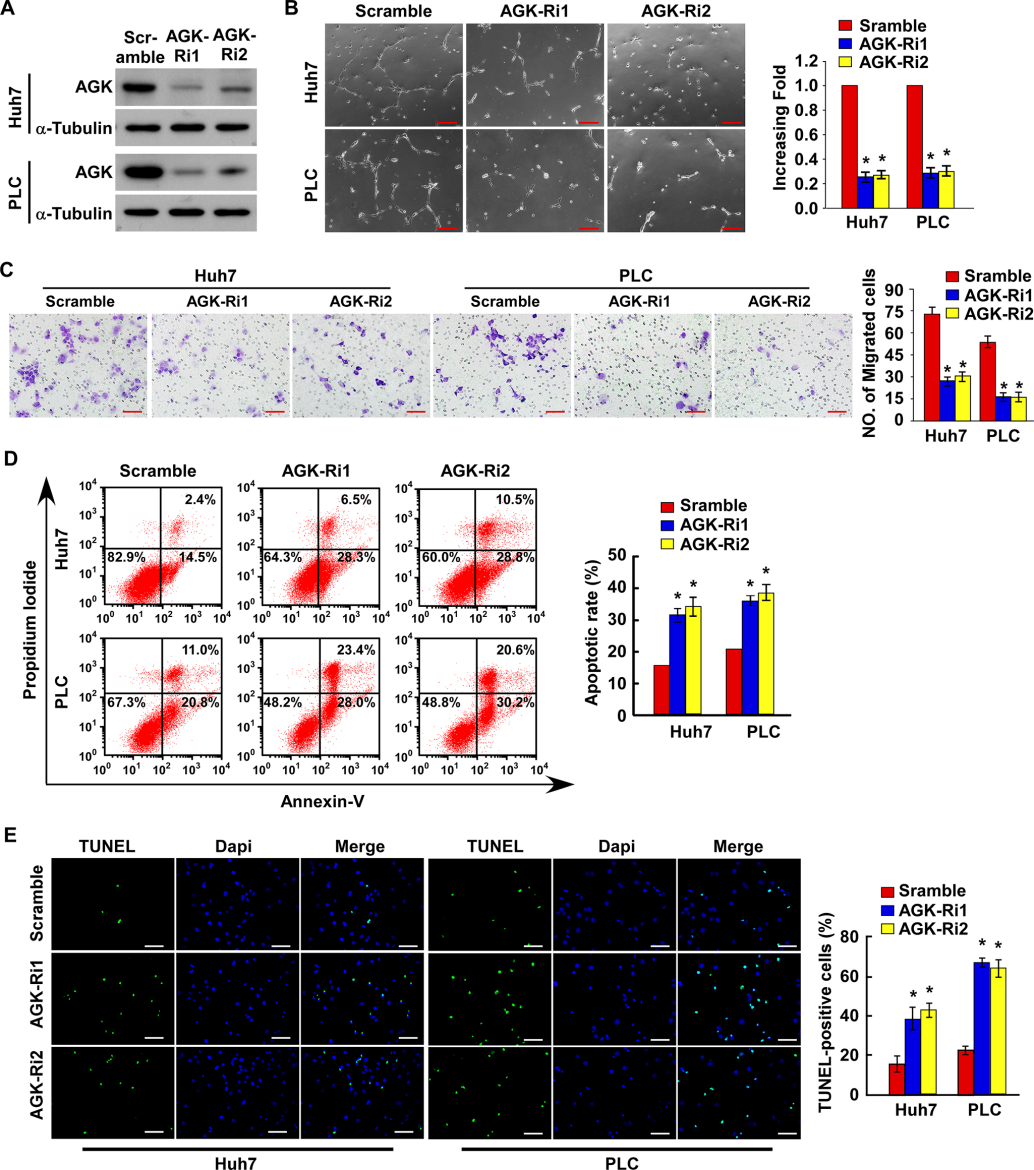
Silencing of AGK inhibits angiogenesis and induces apoptosis in HCC cells *in vitro* **(A)** Confirmation of the silencing of *AGK* by two specific short hairpin RNAs in Huh-7 and PLC HCC cells; α-tubulin was used as a loading control. **(B)** Representative images (left) and quantification (right) of tubule formation by HUVECs cultured in Matrigel-coated plates with conditioned medium collected from the indicated HCC cells. Scale bars: 200 μm. Each bar represents the mean ± SD of three independent experiments; *P < 0.05. **(C)** Cell migration was assessed by culturing HUVECs with conditioned media collected from the indicated HCC cells. Scale bars: 50 μm. Each bar represents the mean ± SD of three independent experiments; *P < 0.05. **(D)** Annexin V-FITC/PI staining of the indicated cells after treatment with cisplatin (20 μM) for 24 hours. Each bar represents the mean ± SD of three independent experiments; *P < 0.05. **(E)** Representative images (left) and quantification of TUNEL-positive cells in the indicated cells after treatment with cisplatin (20 μM) for 24 hours. Scale bars: 50 μm. Each bar represents the mean ± SD of three independent experiments; *P < 0.05.

Conversely, silencing of AGK significantly reduced the ability of HCC cells to induce tubule formation and migration by HUVECs, and increased the sensitivity of HCC cells to cisplatin-induced apoptosis *in vitro* (Figure 3A-E and Supplementary Figure 2A). Taken together, these results suggest that AGK plays an important role in promoting the aggressive behavior of HCC cells.

### AGK contributes to the progression of HCC *in vivo*

**Figure 4:**
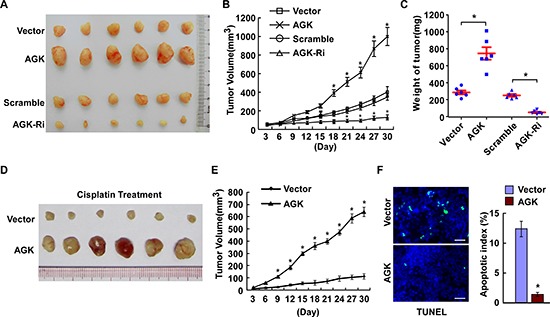
AGK contributes to the progression of HCC *in vivo* **(A)** Representative images of tumors from all of the mice in each group. **(B)** Tumor volumes were measured on the indicated days. **(C)** Mean tumor weights for each group. **(D-F)** Xenograft tumors from the mice treated with cisplatin. **(D)** Images of the tumors from all mice in each group. **(E)** Tumor volumes were measured on the indicated days. **(F)** Apoptotic index, determined by the percentage of TUNEL-positive cells. Scale bars: 50 μm. Each bar represents the mean ± SD of three independent experiments; *P < 0.05.

Next, we investigated the effect of AGK on the tumorigenicity of HCC cells using an in *vivo* mouse model. As shown in Figure 4A-C and Supplementary Figure 2B, the tumors formed by AGK-transduced HCC cells grew more rapidly and were larger in size, while the tumors formed by AGK-silenced cells were smaller in both size and weight, compared to the tumors formed by control cells. Moreover, the tumors established using AGK-transduced HCC cells were resistant to apoptosis and formed larger tumors than control cells in mice treated with cisplatin (Figure 4D-E), and had fewer TUNEL-positive apoptotic cells compared to control tumors (Figure 4F). Collectively, these results indicate that AGK contributes to the progression of HCC *in vivo*.

### AGK regulates the NF-κB signaling pathway in HCC

**Figure 5:**
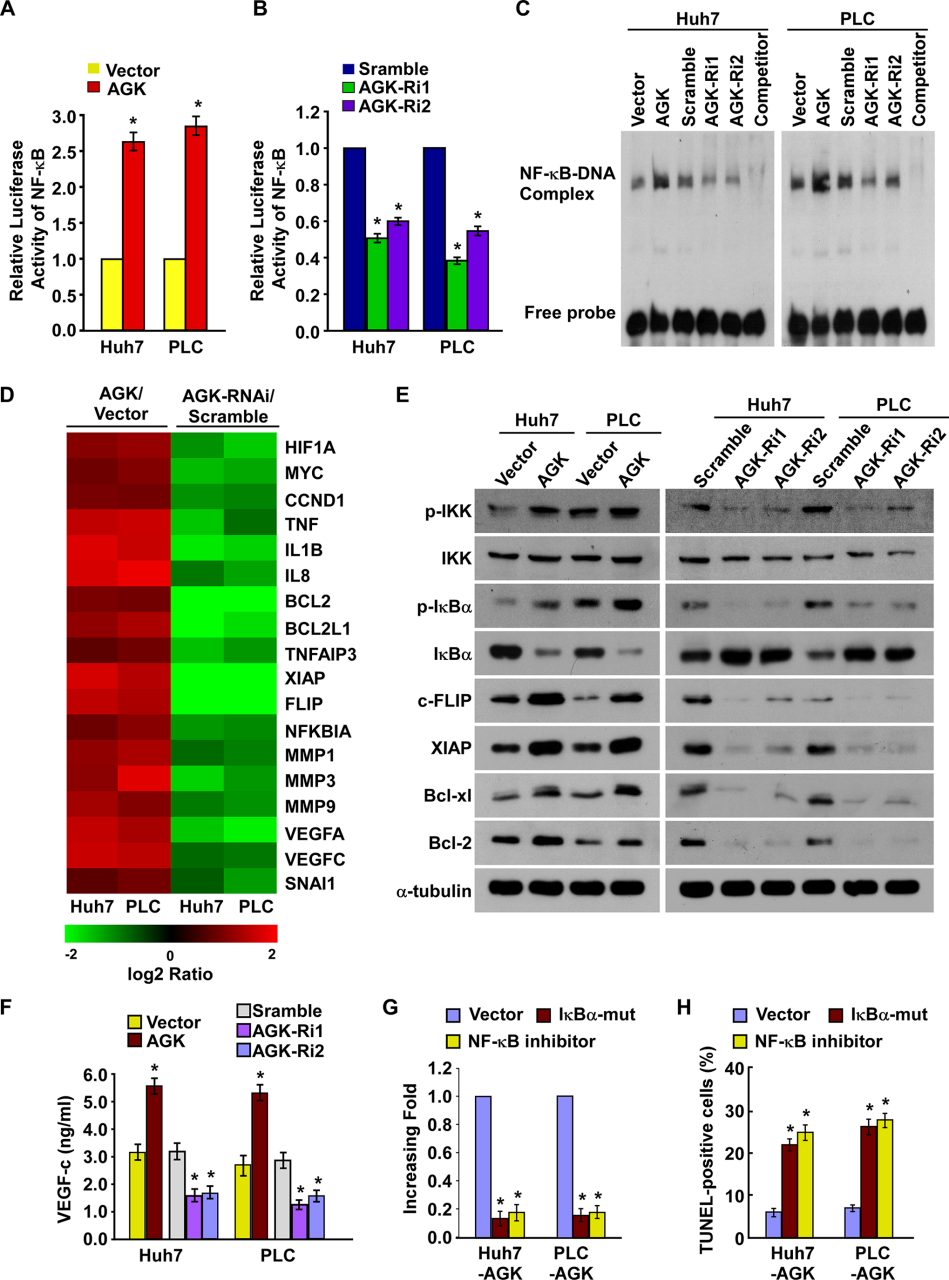
AGK activates the NF-κB signaling pathway **(A)** Activity of the NF-κB luciferase reporter gene in HCC cells expressing either the empty vector or AGK. **(B)** Activity of the NF-κB luciferase reporter gene in HCC cells transfected with *AGK* siRNAs or control vector. Each bar represents the mean ± SD of three independent experiments; *P < 0.05. **(C)** EMSA indicating that NF-κB activity increased in AGK–transduced cells and decreased in AGK–silenced cells. **(D)** Real-time PCR analysis demonstrating an apparent overlap between NF-κB–dependent gene expression and AGK–regulated gene expression. The pseudocolour represents the intensity scale for AGK versus the vector or AGK siRNA versus control siRNA, calculated by log^2^ transformation. **(E)** Western blotting of the expression levels of the indicated proteins in the indicated cells. **(F)** ELISA showing the expression of VEGF-C in AGK–transduced or *AGK*-silenced HCC cells. Each bar represents the mean ± SD of three independent experiments; *P < 0.05. **(G)** Quantification of tubule formation by HUVECs cultured in Matrigel-coated plates with conditioned media from HCC cells transfected with the vector, IκBα-mut or treated with the NF-κB inhibitor (JSH-23). **(H)** Quantification of cisplatin-induced (20 μM) TUNEL-positive cells in HCC cells transfected with vector, IκBα-mut or treated with the NF-κB inhibitor. Each bar represents the mean ± SD of three independent experiments; *P < 0.05.

As a growing body of evidence demonstrates that the NF-κB signaling pathway plays a central role in both angiogenesis and resistance to apoptosis in HCC [28–30], we investigated whether AGK is involved in regulation of the NF-κB signaling pathway in HCC. As shown in Figure 5A-D, overexpression of AGK significantly increased and silencing of AGK reduced the luciferase reporter gene activity, NF-κB DNA-binding ability and mRNA expression levels of numerous well-characterized NF-κB downstream genes, suggesting that AGK may contribute to activation of NF-κB. Moreover, western blotting demonstrated that the phosphorylation of IKK-β and IκBα and the expression of c-FLIP, XIAP, Bcl-xl and Bcl-2, all of which are all well-characterized NF-κB downstream genes, were upregulated in AGK-overexpressing cells and downregulated in AGK-silenced cells (Figure 5E). Additionally, the expression of IκBα was reduced in AGK-transduced cells and increased in AGK-silenced cells (Figure 5E). Furthermore, overexpression of AGK significantly elevated while knockdown of AGK reduced the expression of VEGF-C in HCC cells (Figure 5F).

Next, we investigated whether activation of NF-κB signaling was required for AGK-induced angiogenesis and resistance to apoptosis in HCC cells. As shown in Figure 5G and H, transfection of an IκBα dominant-negative mutant (IκBα-mut) or treatment with a NF-κB inhibitor abrogated the ability of AGK-overexpressing cells to promote tubule formation by HUVECs and reduced the ability of HCC cells to resist apoptosis induced by cisplatin. Taken together, these results indicate that the biological effects of AGK during the progression of HCC are mainly exerted via functional activation of the NF-κB signaling pathway.

### Clinical relevance of AGK-induced NF-κB activation in human HCC

**Figure 6:**
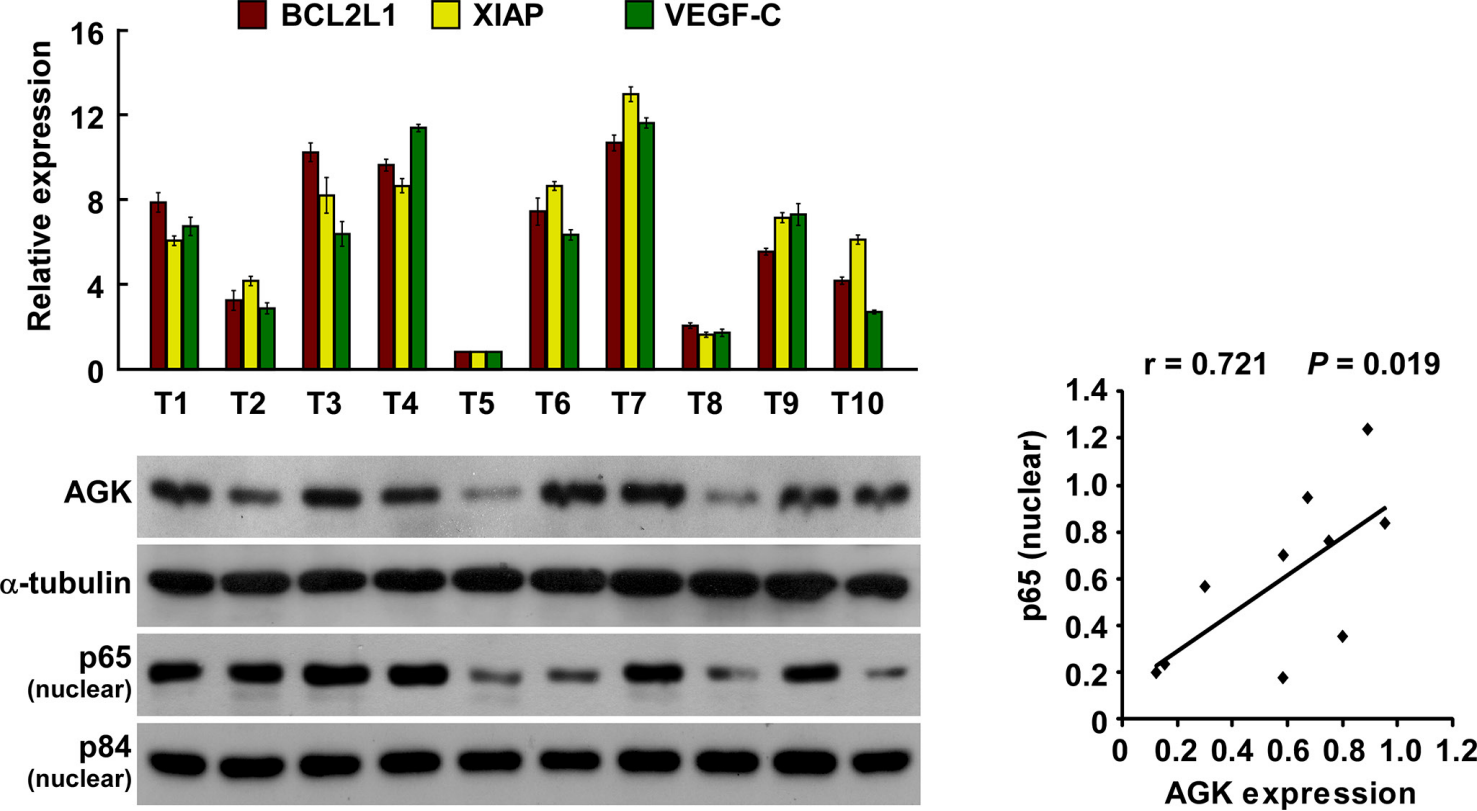
Clinical relevance of AGK-induced NF-κB activation in HCC Expression analysis (left) and correlation (right) of AGK expression and BCL2L1, XIAP, VEGF-C and nuclear p65 expression in 10 freshly collected human HCC tissue samples (T); α-tubulin and nuclear protein p84 were used as loading controls. Each bar represents the mean ± SD of three independent experiments.

We further investigated whether AGK expression and NF-κB activation are clinically relevant in human HCC. As shown in Figure 6, the level of AGK expression in ten freshly collected clinical HCC samples correlated positively with nuclear p65 expression (*r* = 0.721, *P* = 0.019) and the mRNA expression levels of the NF-κB downstream genes BCL2-like 1 (*BCL2L1*; *r* = 0.927, *P* < 0.001), X-linked IAP (*XIAP*; *r* = 0.867, *P* = 0.001) and *VEGF-C* (*r* = 0.758, *P* = 0.011). These data further support the notion that AGK can activate the NF-κB signaling pathway, and in turn may promote angiogenesis and lead to a poor clinical outcome in HCC.

## DISCUSSION

HCC is a hypervascular tumor type with high levels of neovascularization, and angiogenesis plays an essential role in the growth and progression of HCC [31–33]. Extensive evidence indicates that VEGF plays a crucial role in promoting angiogenesis in HCC [34, 35]. Tumor expression of VEGF has been significantly associated with tumor progression and poorer prognosis in patients with HCC [36–38]. Moreover, a high pretreatment serum VEGF level was predictive of poorer response and survival in patients with HCC undergoing chemoembolization [39, 40]. Despite this strong clinical relevance, the precise mechanisms that regulate angiogenesis and VEGF expression in HCC remain poorly clarified. In this study, we demonstrated that overexpression of AGK enhanced, whereas silencing of AGK suppressed, VEGF expression and angiogenesis in HCC cells *in vitro*. More importantly, overexpression of AGK promoted, while silencing of AGK inhibited, the growth and tumorigenicity of HCC cells in an *in vivo* mouse model. Clinical analysis also indicated that high expression of AGK was associated with poorer overall survival in patients with HCC. Therefore, these findings not only reveal a mechanism that promotes angiogenesis in HCC, but also provide insight into novel molecular targets for antiangiogenic therapy in HCC.

One distinguishing feature of HCC compared to other human cancers is its significant association with cirrhosis in the majority of cases. Several lines of evidence have shown that VEGF is upregulated in cirrhotic tissues and VEGF appears to play an important role in angiogenesis and the pathogenesis of cirrhosis, which may explain the pre-malignant potential of cirrhosis [41–43]. In agreement with these observations, analysis of previously published data revealed that *AGK* is significantly upregulated in cirrhotic tissues, which further supports our findings that AGK could upregulate expression of VEGF and promote angiogenesis. However, the biological role of AGK in cirrhosis and its relationship with hepatocarcinogenesis in cirrhotic tissues require further investigation.

Although surgical intervention is considered to be one of the most effective therapeutic strategies, the majority of patients with HCC who are diagnosed at a later stage are ineligible for surgical treatment and mainly receive systemic chemotherapy regimens [44–46]. Multiple chemotherapeutic agents including cisplatin are commonly used for the treatment of HCC; however, these approaches only provide very limited survival advantages due to the significant chemoresistance of HCC cells [47–49]. Thus, it is imperative to identify the key factors that regulate chemoresistance, as this may provide novel therapeutic targets for HCC. Herein, we found that overexpression of AGK enhanced the resistance of HCC cells to cisplatin-induced apoptosis, whereas silencing AGK increased the sensitivity of HCC cells to cisplatin-induced apoptosis; these effects were mediated via modulation of the expression of multiple anti-apoptotic factors including c-FLIP, XIAP, Bcl-xl and Bcl-2. Moreover, an *in vivo* mouse model showed that AGK-transduced HCC cells formed larger tumors than control cells, even when the mice were treated with cisplatin, further confirming the anti-apoptotic effect of AGK. Consistent with our current findings, a previous study demonstrated that AGK was a significant factor that determined the patient response to chemoradiotherapy in esophageal squamous cell carcinoma [26]. Thus, we propose that AGK may have potential as target for therapy in a range of tumor types.

AGK has been reported to be upregulated in various types of tumors including prostate cancer, esophageal squamous cell carcinoma, ovarian cancer, gastric cancer and breast cancer [23, 25, 26]. Combined with the results of our study that AGK expression is elevated in HCC, we may infer that the upregulation of AGK could be a marker for cancerous state and may serve as a universal prognostic factor for cancer. Although evidence has established that NF-κB is constitutively activated in HCC [50], we did not observe significant alteration of AGK in the HCC cells transfected with IκBα-mut or treated with NF-κB inhibitor II. Interestingly, we found that *AGK* locus is located at the same region as *BRAF*, which is a putative proto-oncogene and has been reported to be frequently amplified in tumors [51, 52], suggesting that AGK overexpression in HCC might be attributed to genomic amplification. Thus, further investigation would be needed to reveal the causes for AGK upregulation in HCC.

Numerous studies have demonstrated that constitutive activation of NF-κB signaling plays an important role in the malignant transformation of hepatocytes and progression of HCC, which indicates that the NF-κB signaling pathway can be considered a potentially useful therapeutic target for HCC [50, 53–55]. However, considering the important role of NF-κB signaling in the normal immune response, direct targeting of the NF-κB pathway during the treatment of HCC is controversial [56]. In this study, ectopic overexpression of AGK significantly increased, whereas silencing AGK decreased, NF-κB activity in HCC cells. Additionally, disruption of NF-κB signaling by transfection of an IκBα dominant-negative mutant or treatment with a NF-κB inhibitor abrogated the ability of AGK-overexpressing cells to promote angiogenesis in HUVECs and reduced the resistance of HCC cells to apoptosis. As AGK is aberrantly expressed in HCC and contributes significantly to constitutive NF-κB signaling activation, targeting AGK may provide an effective strategy for inhibiting NF-κB signaling - and its subsequent effects on angiogenesis and resistance to apoptosis - during the treatment of HCC.

In summary, this study provides the first report of the expression of AGK in HCC cell lines and tissues, and demonstrates that upregulation of AGK may contribute to the progression and poor prognosis of human HCC. Additionally, this data suggests that *AGK* may function as an oncogene in HCC and may represent a promising prognostic biomarker for HCC. This study not only further elucidates the precise role of AGK in the pathogenesis of HCC, but also provides new insight into the biological basis of cancer progression and may help to identify more effective therapeutic strategies for HCC.

## MATERIALS AND METHODS

### Cell lines and cell culture

The hepatocellular carcinoma cell lines, including Huh7, Bel7404, PLC/PRF-5, HCC9810, Bel7402, SMMC7721, QGY7701, HepG2215, HepG2, Hep3B and QGY7703, were purchased from the American Type Culture Collection (ATCC, Manassas, VA, USA) and cultured in Dulbecco's modified Eagle's medium (Invitrogen, Carlsbad, CA, USA) supplemented with 10% fetal bovine serum (Invitrogen), 100U/ml penicillin and 100 μg/ml streptomycin (Invitrogen). Normal liver epithelial cells LO2 were purchased from the Chinese Academy of Sciences Committee Type Culture Collection cell bank and were cultured under the conditions stated by the manufacturer. Normal liver epithelial cell THLE-3 were purchased from the American Type Culture Collection (ATCC, Manassas, VA) and cultured as suggested by the manufacturer.

### Patient information and tissue specimens

A total of 245 paraffin-embedded, archived clinical HCC specimens, histopathologically diagnosed at the Sun Yat-sen University Cancer Center from 2002 to 2005, were used in the current study. Prior patient consents and approvals from the Institutional Research Ethics Committee were obtained for the use of these clinical materials for research purposes. Clinical information of the samples is summarized in the Supplementary Table 1. The fresh HCC tissues and the matched adjacent noncancerous tissues (n = 8) and HCC tissues (without matched adjacent noncancerous tissues; n = 10) were frozen and stored in liquid nitrogen until further use.

### Plasmids and transfection

The plasmids for AGK ectopic overexpressing and silencing were constructed as previous study [25]. pNF-κB-luc and control plasmids (Clontech) were used to determine NF-κB activity. pBabe-Puro-IκBα-mut (plasmid 15291) expressing IκBα dominant-negative mutant (IκBα-mut) was purchased from Addgene (Cambridge, MA). Transfection was performed using the Lipofectamine 2000 reagent (Invitrogen, Carlsbad, CA) according to the manufacturer's instruction. Stable cell lines expressing AGK or AGK shRNAs were selected with 0.5 μg/ml puromycin for 10 days.

### RNA extraction and real-time quantitative PCR

Total RNA from cultured cells was extracted using Trizol reagent (Invitrogen, Carlsbad, CA) according to the manufacturer's manual. cDNA was synthesized by M-MLV reverse transcriptase (Promega, Fitchburg, WI). Real-time PCR was performed in ABI Prism 7500 Sequence Detection System (Applied Biosystems, Foster City, CA) using dye SYBR Green I (Roche, Penzberg, Bavaria, Germany). Expression data were normalized to the geometric mean of housekeeping gene *GAPDH* to control the variability in expression levels and calculated as 2^−[(*C_t_* of gene) − (*C_t_* of *GAPDH*)]^, where C*_t_* represents the threshold cycle for each transcript.

### Immunohistochemistry (IHC)

IHC analysis was performed in 245 clinical HCC tissue sections as previously described [25]. The sections were reviewed and scored independently by two observers based on both the proportion of positively stained tumor cells and the intensity of staining. The proportion of positive tumor cells was scored as follows: 0, no positive tumor cells; 1, <10%; 2, 10%–35%; 3, 35%–75%; 4, >75%. The intensity of staining was graded according to the following criteria: 1, no staining; 2, weak staining (light yellow); 3, moderate staining (yellow–brown); 4, strong staining (brown). The staining index (SI) was calculated as staining intensity score × proportion of positive tumor cells. Using this method of assessment, the expression of AGK was scored as 0, 2, 3, 4, 6, 8, 9, 12 and 16. Cut-off values were chosen on the basis of a measure of heterogeneity with the log-rank test statistical analysis with respect to overall survival. SI ≥8 was defined as high AGK expression and SI <8 was defined as low AGK expression.

### Western blotting analysis

Western blotting was performed as previously described [57], using anti-AGK antibody (Epitomics, Burlingame, CA), anti-IKKβ, anti-p-IKKβ (Santa Cruz Biotech., Santa Cruz, CA), anti- IκBα, anti-p-IκBα, anti-c-FLIP, anti-XIAP, anti-Bcl-xl, anti-Bcl-2, anti-p65, anti-p84 antibodies (Cell Signaling, Danvers, MA). The membranes were stripped and re-probed with an anti-α-tubulin antibody (Sigma, Saint Louis, MI) as a loading control.

### Pharmacological inhibitors

NFκB inhibitor II JSH-23 (30 μM; EMD, La Jolla, CA) was dissolved in Me_2_SO (cell culture grade; Sigma) and used to treat cells. Cisplatin (20 μM; Sigma, Saint Louis, MO, USA) was dissolved in 0.9% NaCl.

### Enzyme-linked immunosorbnent assay (ELISA)

The concentration of VEGF-C in the cell conditioned medium was determined by a commercially available ELISA Kit (Calbiochem/Oncogene, Cambridge, MA, USA). Briefly, the collected condition medium was added to a well coated with VEGF-C polyclonal antibody and then immunosorbented with biotinylated monoclonal antihuman VEGF-C antibody at room temperature for 2 h. The color development catalyzed by horseradish peroxidase was terminated with 2.5 M sulphuric acid and the absorption was measured at 450 nm. The protein concentration was calculated by comparing the relative absorbance of the samples with the standards.

### Luciferase assay

Cells (3 × 10^4^) were seeded in triplicates in 48-well plates and allowed to settle for 24 h. 0.1 μg of pNF-κB-luc plasmid, or the control-luciferase plasmid, plus 1 ng of pRL-TK renilla plasmid (Promega), were transfected into HCC cells using the Lipofectamine 2000 reagent (Invitrogen). 48 hours after transfection, luciferase and renilla activities were measured using the Dual Luciferase Reporter Assay Kit (Promega).

### Migration assay

HUVECs (2 × 10^4^) were plated in the upper chamber of the BioCoat^TM^ Invasion Chambers (BD, Bedford, MA) and incubated with the conditional medium collected from HCC cells infected with Vector, AGK or Scramble, AGK-RNAi at 37°C for 22 hours, followed by removal of cells inside the upper chamber with cotton swabs. Migratory cells on the lower membrane surface were fixed in 1% paraformaldehyde, stained with hematoxylin, and counted (Ten random 200 × fields per well). Cell counts are expressed as the mean number of cells per field of view. Three independent experiments were performed and the data are presented as mean ± standard deviation (SD).

### HUVEC tube formation assay

HUVEC tube formation assay was performed as previously described [58]. Briefly, 200 μl of pre-cooled Matrigel (Collaborative Biomedical Products) was transferred into each well of a 24-well plate and polymerized for 30 minutes at 37°C. HUVECs (2 × 10^4^) in 200 μl of conditioned medium were added to each well and incubated at 37°C, 5% CO_2_ for 20 hours. The capillary tube structure was photographed under a 100 × bright-field microscope, and quantified by measuring the total length of the completed tubes. Each condition was assessed in triplicate.

### Electrophoretic mobility shift assay (EMSA)

EMSA was performed as previous study using the LightShift Chemiluminescent EMSA kit (Pierce Biotechnology) [58]. The following DNA probes containing specific binding sites were used: NF-κB sense, 5′-AGTTGAGGGGACTTTCCCAGGC-3′;  NF-κB antisense, 5′-GCCTGGGAAAGTCCCCTCAAC-3′.

### Xenografted tumor mode

4–5 week old BALB/c-nu mice were purchased from the Center of Experimental Animal of Guangzhou University of Chinese Medicine (Guangzhou, China). All experimental procedures were approved by the Institutional Animal Care and Use Committee of Sun Yat-sen University. The BALB/c nude mice were subcutaneous injection of Huh7/Vector, Huh7/AGK or Huh7/Scramble, Huh7/AGK-RNAi cells (5 × 10^6^) in the dorsal flank. Xenograft tumors were examined once every three days. Tumor size was measured using a slide caliper and tumor volume was determined by the formula (L × W^2^)/2 (L indicates the length-diameter and W the width-diameter of the tumor). 30 days after injection, tumors were excised, weighed, and paraffin embedded. Serial 4.0-μm sections were cut and stained with haematoxylin and eosin according to standard protocols. The apoptotic index was measured by assessing the percentage of TUNEL-positive cells. In the experiment testing the chemoresistent effect of AGK, the BALB/c nude mice (4–5 weeks of age, 18–20 g; *n =* 6/group) were implanted subcutaneously with Huh7/vector and Huh7/AGK cells (5 × 10^6^, suspended in 100 μl sterile PBS). After 6 days, animals were intraperitoneally injected with 100 μl cisplatin (6mg/kg) every other day. On day 30, animals were euthanized, and tumors were excised, weighed and subjected to pathological examination.

### Statistical analysis

All statistical analyses were carried out using the SPSS13.0 statistical software package. The chi-square test was used to analyze the relationship between AGK expression and clinicopathological characteristics. Survival curves were plotted by the Kaplan–Meier method and compared using the log-rank test. Comparisons between groups for statistical significance were carried out with a 2-tailed paired Student's t-test. In all cases, *P* < 0.05 was considered significant.

## SUPPLEMENTARY FIGURES AND TABLE


