# Supplementary material for: AGK enhances angiogenesis and inhibits apoptosis via activation of the NF-κB signaling pathway in hepatocellular carcinoma
Source: Oncotarget. 2014 Nov 29;5(23):12057–69. doi: 10.18632/oncotarget.2666 (PMC4323001; doi:10.18632/oncotarget.2666)
Supplement: Supplementary file 1 [file oncotarget-05-12057-s001.pdf]

SUPPLEMENTARY FIGURES AND TABLE

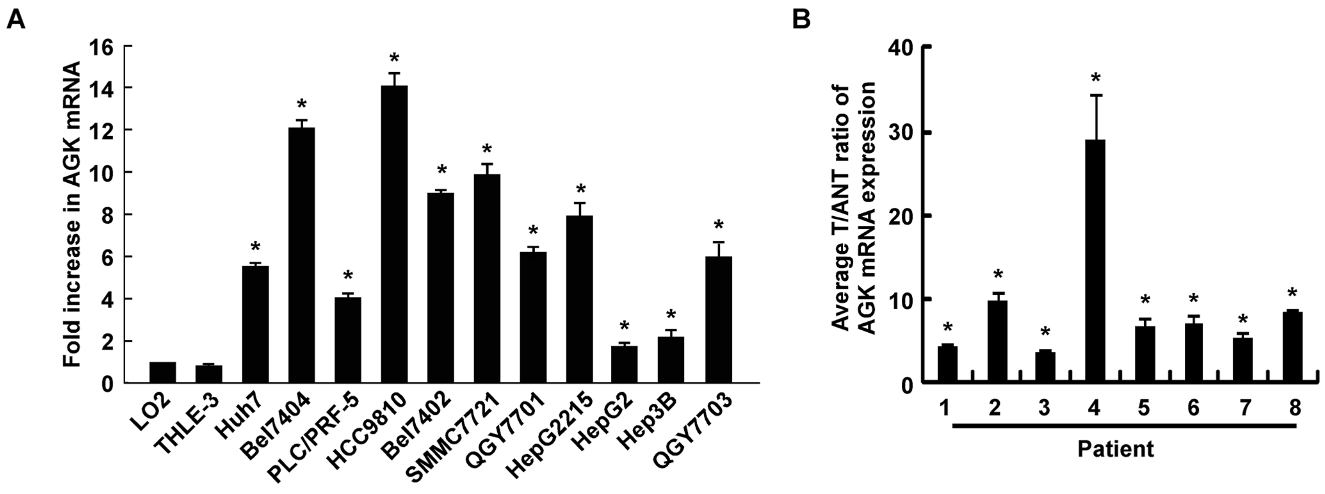

Supplementary Figure S1: Real-time PCR analysis of AGK mRNA expression in HCC cell lines **(A)** and eight paired HCC and tumor adjacent liver tissues **(B)**. *$P < 0.01$.

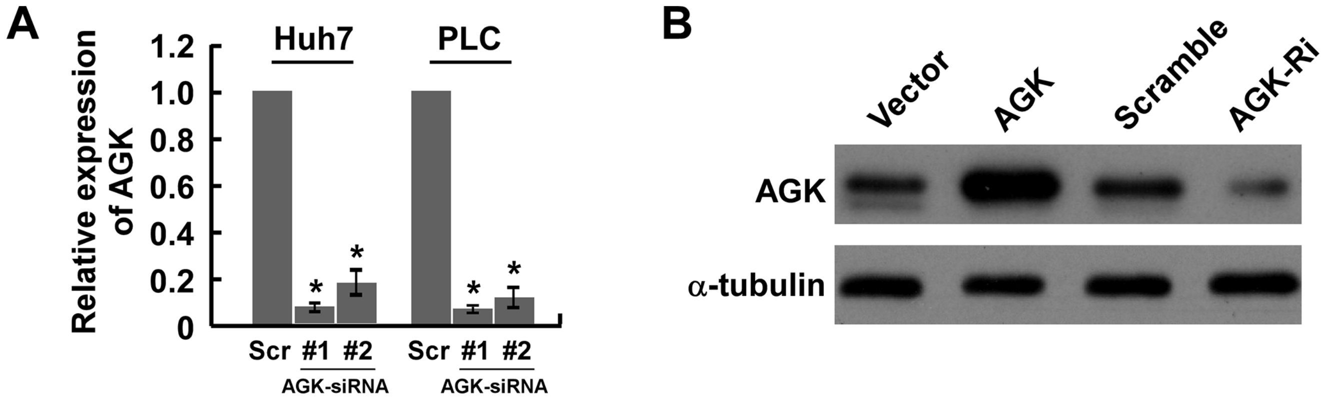

Supplementary Figure S2: (A) Quantification of the silencing effect of AGK by two specific short hairpin RNAs in Huh7 and PLC HCC cells. Each bar represents the mean ± SD of three independent experiments; *$P < 0.01$. **(B)** Western blotting analysis of AGK expression in xenografts formed from Huh7/Vector, Huh7/AGK or Huh7/Scramble or Huh7/AGK-RNAi cells.

Supplementary Table S1. Correlation between Clinicopathologic Features and AGK expression levels

Characteristics		Total (n = 245)	AGK		χ^2 test *p*-value	Fisher's test *p*-value
			Low expression	High expression		
Age (y)	≥ 48	129	63	66	0.558	0.609
	< 48	116	61	55		
Gender	Male	220	107	113	0.066	0.090
	Female	25	17	8		
Clinical stage	I	25	19	6	0.001	< 0.001
	II	94	55	39		
	III	123	50	73		
	IV	3	0	3		
T classification	T_1	25	19	6	0.002	0.001
	T_2	107	60	47		
	T_3	102	43	59		
	T_4	11	2	9		
N classification	N_0	231	117	114	0.962	1.000
	N_1	14	7	7		
M classification	Yes	3	0	3	0.078	0.119
	No	242	124	118		
AFP	≥ 400	69	40	29	0.149	0.158
	< 400	176	84	92		
